# Pharmacological activation of SIRT1–AMPK by ginsenoside Rb1: a novel therapeutic strategy for pressure injury *via* dual suppression of ferroptosis and inflammation

**DOI:** 10.3389/fphar.2025.1683479

**Published:** 2026-02-17

**Authors:** Hongbo Zhu, Hang Li, Yinong Shi, Hua Zhi, Xuelu Zhao, Zhiwen Wang, Jinhui Liu

**Affiliations:** 1 Department of Surgery, Affiliated Hospital of Hebei University of Engineering, Handan, China; 2 Procurement Office, Affiliated Hospital of Hebei University of Engineering, Handan, China

**Keywords:** ferroptosis, ginsenoside Rb1, multi-omics analysis, oxidative stress, pressure injury, sirtuin 1–AMP-activated protein kinase pathway

## Abstract

**Background:**

Pressure injuries (PIs) are a major clinical problem, and current treatments offer limited efficacy. Ferroptosis-driven oxidative damage and chronic inflammation severely impair wound healing. Ginsenoside Rb1 (Rb1), a bioactive component of *Panax ginseng*, possesses antioxidant and anti-inflammatory activities, yet its therapeutic potential in PI via ferroptosis regulation has not been investigated. This study aims to determine whether Rb1 promotes PI wound repair by activating the Sirtuin 1–AMP-activated protein kinase (SIRT1–AMPK) pathway to inhibit ferroptosis and inflammation, thereby providing a new pharmacological strategy for PI management.

**Method:**

Transcriptomic profiling was performed on dorsal skin tissues from normal rats, pressure injury rats, and Rb1-treated rats using RNA sequencing to identify differentially expressed genes (DEGs), followed by Gene Ontology (GO)/Kyoto Encyclopedia of Genes and Genomes (KEGG) enrichment, network pharmacology analysis, and protein–protein interaction (PPI) network construction to screen potential regulatory pathways. *In vitro*, ferroptosis was induced in an L929–HaCaT co-culture system using erastin/RSL3, and cells were treated with various concentrations of Rb1. Cell viability, reactive oxygen species (ROS) levels, ferroptosis-related markers (GPX4, SLC7A11, and ACSL4), and SIRT1–AMPK pathway proteins were evaluated by using the CCK-8, assay, fluorescence assays, Western blotting, and RT-qPCR. *In vivo*, a PI model was created in Sprague–Dawley (SD) rats, followed by administration of Rb1. Wound healing, histopathology, oxidative stress indices, inflammatory cytokines, and SIRT1–AMPK activation were assessed.

**Results:**

Integrated transcriptomic and network pharmacology analyses identified the SIRT1–AMPK axis as a key mediator of Rb1-induced wound repair. *In vitro*, Rb1 dose-dependently attenuated erastin/RSL3-induced ferroptosis, decreased ROS levels, and increased the expressions of GPX4, SLC7A11, and ACSL4, while simultaneously activating SIRT1 and downstream p-AMPK. Rescue experiments showed that blocking the activities of SIRT1 or AMPK diminished the protective effects of Rb1 against ferroptosis. *In vivo*, high-dose Rb1 accelerated wound closure, activated SIRT1–AMPK signaling, enhanced ferroptosis-related protein expression, and reduced tumor necrosis factor-α (TNF-α) and interleukin-6 (IL-6) levels.

**Conclusion:**

Rb1 functions as an SIRT1–AMPK activator that inhibits ferroptosis and inflammation to promote PI wound healing. These findings underpin the efficacy of Rb1 as a promising multi-target therapeutic candidate for future clinical development.

## Graphical Abstract

**Figure:**
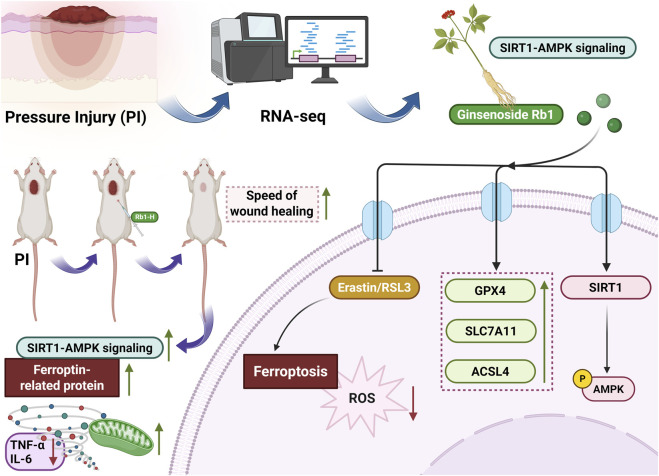
Transcriptomic analysis reveals the molecular mechanism by which the herbal compound Rb1 promotes PI wound regeneration by activating SIRT1-mediated AMPK energy metabolism signaling and inhibiting ferroptosis.

## Introduction

Pressure injury (PI), also referred to as pressure ulcer, represents a localized area of tissue ischemia and necrosis caused by exposure to prolonged pressure, shear forces, or friction on the skin and underlying tissues. PI constitutes a common and refractory chronic wound in clinical practice (Gillespie et al., 2020). High incidence of PI is observed among individuals who are bedridden, have restricted mobility, or present with impaired consciousness, with elderly patients, individuals with spinal cord injury, and those in intensive care units being particularly vulnerable (Guihan and Richardson, 2018; Sgarzani et al., 2023). According to the Wound Healing Society, PI is characterized by delayed wound closure, persistent inflammation, tissue necrosis, and chronic pain, leading to a substantial decline in the quality of life and increasing the risk of life-threatening complications such as sepsis (Qaseem et al., 2015; Snyder and Lanier, 2002). Current therapeutic approaches, such as pressure offloading, debridement, wound dressing, infection control, and nutritional support, have produced few clinical benefits (Shevchenko et al., 2021; Xu et al., 2017). A major barrier to effective healing lies in sustained cellular dysfunction, oxidative stress, and unresolved inflammation within the wound microenvironment (Xu et al., 2024; Wall et al., 2008). Therefore, clarification of the underlying pathophysiology of PI, especially the mechanisms of regulated cell death, may promote the development of more precise therapeutic strategies.

Ferroptosis, a distinct form of regulated cell death driven by iron-dependent lipid peroxidation, has gained increasing attention within the field of wound healing (Zhao et al., 2025). It is characterized by decreased activity of glutathione peroxidase 4 (GPX4), elevated intracellular iron levels, and accumulation of reactive oxygen species (ROS), which have been widely implicated in tissue injury in neurodegeneration, cancer progression, and organ damage (Yin et al., 2024; Bi et al., 2023). Evidence from chronic wound models indicates that ferroptosis undermines the proliferative capacity of fibroblasts and keratinocytes, promotes oxidative damage, and amplifies inflammatory responses, thereby impairing wound repair (Jiang et al., 2025; Feng et al., 2022). Although previous research has studied ferroptosis in diabetic ulcers and burn wounds, its role in the pathogenesis of PI remains insufficiently defined (Feng et al., 2025). Frequent detection of iron accumulation and elevated ROS levels in PI lesions suggests the central contribution of ferroptosis to disease progression (Ishikawa et al., 2024). Identification of molecular regulators and signaling pathways controlling ferroptotic injury may provide opportunities for targeted intervention and improved PI management.

Sirtuin 1 (SIRT1), a member of the NAD^+^-dependent deacetylase family, is a key energy sensor and stress regulator involved in cellular metabolism, aging, inflammation, and oxidative stress responses (Kitada et al., 2019; Tao et al., 2023). Activation of AMP-activated protein kinase (AMPK), a central intracellular energy sensor, promotes metabolic homeostasis and alleviates mitochondrial dysfunction and oxidative stress (Wu and Zou, 2020; Carvalho et al., 2025). Growing evidence indicates a cooperative interaction between SIRT1 and AMPK, forming a regulatory axis that confers protective effects across multiple tissue injury models (Li et al., 2024; Zhao et al., 2018). Mechanistically, SIRT1 activates AMPK by deacetylating LKB1, thereby enhancing antioxidant gene expression, preserving mitochondrial integrity, and inhibiting apoptosis and ferroptosis (Liu C.-Y. et al., 2021; Yang et al., 2025). However, systematic investigations into the roles of the SIRT1–AMPK pathway in PI and in ferroptosis regulation remain limited (Zhang et al., 2025). Clarification of whether activation of this signaling axis can reduce ferroptotic injury may provide new therapeutic opportunities for PI management.

Traditional Chinese medicine (TCM), characterized by multi-target, multi-pathway regulation, has long shown therapeutic potential in chronic wound repair (Zhang et al., 2021). Ginsenoside Rb1 (Rb1), a major bioactive constituent of *Panax ginseng*, exhibits strong antioxidant, anti-inflammatory, anti-apoptotic, and pro-proliferative activities (Liu et al., 2022; Zhou et al., 2024). Previous studies have demonstrated that Rb1 promotes tissue regeneration through multiple mechanisms, including enhanced wound healing in diabetes, neuroprotection after ischemia–reperfusion injury, and preservation of cardiomyocyte viability (He et al., 2024; Chen S. et al., 2019). These effects are mediated through modulation of oxidative stress, apoptosis, and cellular energy metabolism (Su et al., 2022; Liu Y. et al., 2021). Additional findings indicate that Rb1 stimulates intestinal epithelial repair through ERK and Rho signaling pathways (Toyokawa et al., 2018), accelerates burn wound healing in diabetic rats by supporting the shift of macrophages from M1 to M2 phenotypes (Venkatesan et al., 2023), and enhances SASP-mediated tissue repair *via* activation of the p38MAPK, MSK2, and NF κB pathways (Hou and Kim, 2018). Evidence evaluating Rb1 activity in PI remains limited, and the involvement of the SIRT1–AMPK signaling pathway and ferroptosis regulation has not been fully elucidated (Zheng et al., 2020; Zhai et al., 2024). A comprehensive research strategy that integrates multi-omics analysis with *in vitro* and *in vivo* experimentations may provide deeper insights into the underlying mechanisms.

This study aims to systematically elucidate the molecular mechanisms by which Rb1 facilitates PI repair, with a particular focus on its ability to activate the SIRT1-mediated AMPK energy metabolism pathway and suppress ferroptosis. Using RNA sequencing (RNA-seq), we identified differentially expressed genes (DEGs), followed by Gene Ontology (GO)/Kyoto Encyclopedia of Genes and Genomes (KEGG) enrichment analyses and protein–protein interaction (PPI) network construction to uncover key signaling nodes. *In vitro*, ferroptosis was induced in L929 fibroblasts and HaCaT keratinocytes using erastin/RSL3, and the effects of Rb1 on cell viability, ROS levels, and ferroptosis-related protein expression were evaluated. *In vivo*, a PI model was established in Sprague–Dawley (SD) rats to assess wound healing dynamics, inflammation, and pathway activation in response to Rb1. Integrated transcriptomic profiling together with molecular and phenotypic validation delineated the regulatory function of Rb1 in inhibiting ferroptosis and enhancing tissue regeneration. These findings offer a basis for identifying new molecular targets and therapeutic approaches for PI management and contribute to the advancement of evidence-based applications of TCM in chronic wound care.

## Materials and methods

### Ethical statement

All animal procedures were carried out in accordance with the established ethical guidelines and regulations for animal experimentation. The study protocol was approved by the Institutional Animal Care and Use Committee (IACUC) (Ethical Approval Number: IACUC-Hebeu-2024-0028, 2024, 06). All animals were housed under standardized conditions and handled humanely, and all experimental interventions were performed with efforts to reduce pain and distress. At study completion, all rats were humanely euthanized using ether anesthesia.

### Transcriptomic sequencing and analysis

Total RNA was extracted from the dorsal skin tissues of normal rats (Normal, *n* = 3) and PI-induced rats (Injury, *n* = 3), along with from PI-induced rats (Injury, *n* = 3) and Rb1-treated pressure injury rats (Treatment, *n* = 3), for subsequent RNA sequencing. Total RNA extraction was performed using TRIzol reagent (Thermo, 16096020, USA), and RNA integrity was evaluated using the Agilent 2100 Bioanalyzer (Agilent Technologies, USA), ensuring the RNA integrity number (RIN) values exceeded 7.0. RNA libraries were constructed using the NEBNext® Ultra™ RNA Library Prep Kit (NEB, E7435L, Beijing, China), compatible with the Illumina® sequencing platform, and then sequenced on the Illumina NovaSeq 6000 system (Illumina, USA) with 150-bp paired-end reads, achieving a sequencing depth of more than 40 million reads per sample.

Raw reads were subjected to quality filtering with fastp (v0.23.2) to remove adapter sequences and low-quality bases. Clean reads were aligned to the *Rattus norvegicus* reference genome (Rnor_6.0) obtained from Ensembl using HISAT2 (v2.2.1). Gene expression levels were quantified using FeatureCounts (v2.0.1) to generate a count matrix. DEGs were identified using DESeq2 (v1.38.3) with Wald’s test. *P*-values were adjusted for multiple testing using the Benjamini–Hochberg method. Significant DEGs were defined as |log_2_fold change| > 0.5 and *p* < 0.05.Venn analysis was performed using the VennDiagram package (v1.7.3). Functional enrichment data for Rb1-interacting genes were retrieved from the Comparative Toxicogenomics Database (CTD) (https://ctdbase.org/), including GO and KEGG pathway annotations.

### GO and KEGG functional enrichment analyses

ClusterProfiler (v4.6.2) was used to analyze the biological functions of the injury-related DEGs, Rb1-DEGs, and 476 DEGs with reversed expression patterns. GO enrichment included biological process (BP), cellular component (CC), and molecular function (MF) components. KEGG pathway data were obtained from the official KEGG database (https://www.genome.jp/kegg/).

A two-tailed hypergeometric test was applied to evaluate enrichment significance, with all detected genes composing the background set. *P*-values were adjusted using the Benjamini–Hochberg method to obtain q-values, with *q* < 0.05 considered statistically significant. Visualization was performed using the dotplot() function.

To quantitatively compare pathway importance, GeneRatio and enrichment factor were calculated and ranked by –log10(q-value). Bubble size indicates the number of enriched genes, and color represents significance levels.

### Least absolute shrinkage and selection operator (LASSO) regression analysis

Key functional genes associated with Rb1 intervention were identified through the construction of a LASSO regression model based on the 476 reverse-expression DEGs. Modeling was performed using glmnet (v4.1-8) with z-score normalization of input variables and sample type (Treatment *versus* Injury). Ten-fold cross-validation (cv.glmnet) was used to determine the optimal λ value corresponding to the minimum mean squared error (MSE, λ.min).

Model stability was evaluated by plotting the MSE–λ curve and calculating the prediction correlation coefficient (*R* ^2^). Genes with non-zero coefficients were selected as feature genes, and their statistical significance was assessed through permutation testing (1,000 resampling iterations).

### PPI network analysis

Potential interactions among Fa2h, Sirt1, and ferroptosis-related genes were examined by constructing a PPI network using the STRING database (v11.5, https://string-db.org/). A total of 21 ferroptosis-associated genes, along with Fa2h and Sirt1, were input into STRING. The minimum required interaction score was set to 0.4. STRING weighted scoring was used to determine the interaction confidence. Isolated nodes were removed before exporting of the final network.

### Cell culture and experimental grouping

Human keratinocytes (HaCaT cells, PCS-200-011) and L929 fibroblasts (CVCL_0462) were obtained from the American Type Culture Collection (ATCC, USA) and maintained in Dulbecco’s modified Eagle medium (DMEM; D0822, Merck, USA) supplemented with 10% fetal bovine serum (FBS; F8687, Gibco, USA) and 1% penicillin–streptomycin (TMS-AB2, Merck, USA). All cultures were incubated at 37 °C in a humidified incubator with 5% CO_2_. All cell culture procedures were conducted under strict aseptic conditions. A Transwell system was used for co-culture of L929 and HaCaT cells. L929 cells (1 × 10^5^/mL) and HaCaT cells (1 × 10^5^/mL) were seeded into the upper and lower chambers, respectively (Wang et al., 2020).

For cell experiments, 10 mM ginsenoside Rb1 (SM6032-10mM, Beyotime, China), a major bioactive constituent of *Panax ginseng*, was used. According to the published protocols (Joh et al., 2011), the low-dose group received 10 μM Rb1 (1 μL of 10 mM stock into 1 mL medium), while the high-dose group received 20 μM Rb1 (2 μL of the stock into 1 mL medium).

The experiments were performed by inducing ferroptosis in HaCaT cells. The cells were assigned to the following groups: Control: no treatment; Ferroptosis: treatment with RSL3 [2 uM, SML2234, Merck, USA (Zheng F. et al., 2021)] to induce ferroptosis; Rb1-L: treatment with a low concentration of Rb1 during ferroptosis induction; Rb1-H: treatment with a high concentration of Rb1 during ferroptosis induction; Fer-1: ferroptosis inhibitor group (2 μM; NBS5879, Shanghai Nonin Biological Technology Co., Ltd.) (Skouta et al., 2014); Rb1-H + si-NC + DMSO: treated with high-dose Rb1 and transfected with the siRNA-negative control, followed by DMSO treatment; Rb1-H + si-SIRT1+ DMSO: treated with high-dose Rb1 and transfected with SIRT1-targeting small interfering RNA (siRNA) to knock down SIRT1 expression, followed by DMSO treatment; Rb1-H + si-SIRT1 + AMPK: in addition to administration of high-dose Rb1 and SIRT1 knockdown, AMPK was activated using 5-aminoimidazole-4-carboxamide ribonucleotide (AICAR) (0.5 mM, 2627-69-2, Sigma-Aldrich, USA) to investigate the role of the SIRT1–AMPK pathway in ferroptosis regulation.

### Cell viability assay (CCK-8)

HaCaT cell viability was assessed using the cell counting kit-8 (CCK 8) (CK04, Dojindo, Japan). Cells were seeded into 96-well plates at a density of 2 × 10^4^ cells per well and allowed to adhere for 24 h before treatment. After a 24-h incubation with the designated treatments, 10 µL of the CCK 8 reagent was added to each well and incubated at 37 °C for 2 h. The absorbance was measured at 450 nm using a microplate reader (Thermo Fisher Scientific, USA). Each experiment was performed in triplicate.

The ell viability was calculated using the formula: cell viability (%) = (OD_exp_ − OD_blank)/(OD_control − OD_blank) × 100%.

### ROS detection

Intracellular ROS levels were assessed using a commercial ROS assay kit (S0033S, Beyotime, China). HaCaT cells were seeded in 6-well plates at 2 × 10^5^ cells per well. After experimental treatments, cells were incubated with 10 µM 2′,7′-dichlorofluorescin diacetate (DCFH-DA) fluorescent probes at 37 °C for 30 min in the dark. Cells were washed three times with phosphate-buffered saline, and fluorescence images were captured using a fluorescence microscope (FV 1000/ES, Olympus, Japan). Quantitative analysis of fluorescence intensity was performed using ImageJ software and reported as relative fluorescence units. Tissue-level ROS analysis was conducted using a tissue-specific detection kit (HR8821, Beijing Baiao Laibo Technology Co., Ltd., China). The wound tissue (50 mg) was homogenized in distilled water using a glass homogenizer. The homogenate was centrifuged at 100 *g* for 3 min at 4 °C, and the supernatant was collected. A 200-µL aliquot was transferred to a 96-well plate and mixed with 2 µL of the DHE probe. Samples were gently pipetted to ensure complete mixing and then incubated at 37 °C for 15–30 min in the dark. Fluorescence intensity was measured using a microplate reader (E8051, Promega, USA) with excitation wavelength at 488–535 nm and emission wavelength at 610 nm. ROS levels were normalized to protein concentration and expressed as relative fluorescence units per milligram of protein. All experiments were conducted in triplicate.

### Cell transfection and gene silencing

SIRT1 gene silencing was performed using siRNA. HaCaT cells were seeded in 6-well plates and cultured to 50%–60% confluence. Transfection was conducted using Lipofectamine 3000 (L3000001, Invitrogen, USA). For each well, 50 nM siRNA and 2 µL transfection reagent were mixed with Opti-MEM medium (51985091, Gibco, USA), incubated for 15 min, and then added to the culture medium. After 48 h of transfection, SIRT1 expression was assessed using real-time quantitative polymerase chain reaction (RT-qPCR) and Western blot analysis to confirm knockdown efficiency. The siRNA sequences were as follows: si-NC: UUC​UCC​GAA​CGU​GUC​ACG​UTT; si-SIRT1-1: GAT​CCA​AGA​CCA​TTC​TTC​AAG​TTT​G; si-SIRT1-2: CAG​GTT​GCG​GGA​ATC​CAA​AGG​ATA​A.

### RT-qPCR for gene expression analysis

Total RNA was extracted from HaCaT cells and wound tissues using TRIzol reagent (A33254, Thermo Fisher Scientific, USA). First-strand cDNA synthesis was carried out using a reverse transcription kit (RR047A, Takara, Japan). Quantitative PCR was performed using PowerUp™ SYBR™ Green Master Mix (A25742, Thermo Fisher Scientific, USA) on an ABI7500 real-time PCR system. The cycling conditions included an initial denaturation at 95 °C for 30 s, followed by 40 cycles of 95 °C for 5 s and 60 °C for 30 s, and a melt curve step at 95 °C for 15 s, 60 °C for 60 s, and 90 °C for 15 s. GAPDH served as the internal control. All reactions were performed in technical triplicate and repeated independently three times. Relative gene expression was calculated using the 2^−ΔΔCT^ method. Primer sequences are listed in Supplementary Table S1.

### Western blot analysis

Total protein from HaCaT cells or wound tissues was extracted using RIPA lysis buffer (89900, Thermo Fisher, USA), and protein concentration was determined using a BCA assay (23225, Thermo Fisher, USA). Samples were denatured in SDS loading buffer at 95 °C–100 °C for 5 min, and 30 μg of the protein was separated using SDS PAGE under a constant voltage of 80 V in 1× SDS running buffer. Proteins were transferred onto PVDF membranes (IPVH00010, Millipore, USA) using a wet transfer system at a constant current of 200 mA for 1–2 h in 1× Tris glycine buffer containing 20% methanol. Membranes were blocked with 5% non-fat milk (LP0033B, Thermo Fisher, USA) and incubated overnight at 4 °C with primary antibodies against ACSL4, GPX4, solute carrier family 7 member 11 (SLC7A11), SIRT1, AMPK, phosphorylated AMPK (p-AMPK), acetyl-coA carboxylase (ACC), phosphorylated ACC (p-ACC), and GAPDH (see Supplementary Table S2 for antibody details). After washing, membranes were incubated with HRP-conjugated anti-rabbit IgG secondary antibodies (2729, Cell Signaling Technology, USA). Protein bands were visualized using an enhanced chemiluminescence substrate (32106, Thermo Fisher, USA) and imaged using a chemiluminescence detection system (Bio Rad, USA). Band intensities were quantified using ImageJ software (v1.52, NIH, USA).

### Establishment of the PI animal model

Male and female SD rats (*n* = 6 per group, 200–250 g) were obtained from Beijing Vital River Laboratory Animal Technology Co., Ltd. Animals were housed under specific pathogen-free (SPF) conditions (22 °C ± 2 °C, 50% ± 10% humidity, and 12-h light/dark cycle) with *ad libitum* access to food and water. All animals were marked using dye-based identification methods. Saturated picric acid solution (yellow), 0.5% neutral red solution (red), and gentian violet solution (purple) were applied with a brush to distinct body surface locations to generate unique identification codes. Markings were applied in a fixed sequence from left to right and from the anterior to posterior part. A standardized PI model was established by applying cyclic mechanical compression (100 mmHg) to the dorsal skin using a custom-built device. Each cycle consisted of 2 h of compression followed by 1 h of reperfusion, and the cycle was repeated three times (Cui et al., 2016).

Ginsenoside Rb1 (100 mg; SM6032-100mg, Beyotime, China) was dissolved in 2 mL of DMSO to create a 100-mg/mL stock solution. Rats in the low-dose group received subcutaneous Rb1 injections at a dose of 2 mg/kg, and rats in the high-dose group received 10 mg/kg. Rb1 (41753-43-9) was also purchased from Macklin (China) for animal use.

Rats were randomly assigned to five groups (n = 6 per group): Sham, with no pressure applied; Model, with PI induction and no treatment; Rb1 L, with low-dose Rb1 administered once daily for 7 days; Rb1 H, with high-dose Rb1 administered once daily for 7 days; and Fer 1, with 10 mg/kg ferrostatin 1 administered subcutaneously once daily for 3 days (Zhang et al., 2023).

### Wound healing assessment and area measurement

Digital images of the wound sites were captured on days 0, 3, 7, 10, and 14 using a Canon EOS 80D camera (Japan). Wound areas were quantified using ImageJ software (v1.53t), and the wound closure rate was calculated as follows: Wound closure (%) = [(initial area − remaining area)/initial area] × 100%. The wound area was measured by outlining the boundary of the newly formed epithelium, identified as the smooth pink tissue at the wound margin, rather than the edge of the scab or eschar.

### Hematoxylin and eosin (H&E) staining

On day 14, rats were euthanized by anesthesia, and wound tissues were collected and fixed in 4% paraformaldehyde (30525-89-4, Sigma-Aldrich, USA) at ambient temperature for 24 h. Tissues were dehydrated through a graded ethanol series, embedded in paraffin, and sectioned at 5 μm. Sections were deparaffinized in xylene, rehydrated through a graded series of alcohols, and stained with hematoxylin for 5 min. After rinsing, sections were differentiated in acid alcohol for 10 s, blued in ammonia water for 10 s, and counterstained with eosin for 2 min. Sections were then dehydrated, cleared in xylene, and mounted. Morphological evaluation was performed using an Olympus BX53 microscope (Olympus, Japan).

### Collagen fiber assessment (Masson’s trichrome staining)

After deparaffinization and rehydration, wound tissue sections were stained using a Masson trichrome staining kit (G1340, Solarbio, China). Collagen fibers appeared blue, muscle fibers red, and nuclei dark blue. Sections were examined using an Olympus BX53 microscope (Olympus, Japan), and the relative collagen area (%) was quantified using ImageJ software.

### Immunohistochemical analysis

Wound tissue sections were deparaffinized, rehydrated, and subjected to heat-mediated antigen retrieval. Endogenous peroxidase activity was blocked by 15-min incubation in 3% hydrogen peroxide, followed by blocking with 5% bovine serum albumin (BSA) for 1 h. Primary antibodies were applied overnight at 4 °C: tumor necrosis factor-α (TNF-α) (1:1,000, ab307164, Abcam, UK), interleukin-6 (IL-6) (1:1,000, ab9324, Abcam, UK), interleukin-10 (IL-10) (1:500, PA5-94918, Thermo Fisher), 4-hydroxynonenal (4-HNE) (1:25, ab48506, Abcam, UK), CD8 (1:500, 14-0084-82, Thermo fisher), CD80 (1:100, PA5-79001, Thermo fisher), and CD163 (1:100, MA5-16656, Thermo fisher). After washing with PBS, sections were incubated with HRP-conjugated secondary antibodies (1:1,000, ab6721, Abcam, UK) at 37 °C for 1 h. Visualization was achieved using diaminobenzidine (DAB) substrate, followed by hematoxylin counterstaining. Images were captured using an Olympus BX53 microscope, and the percentage of positively stained areas was quantified using ImageJ.

### Enzyme-linked immunosorbent assay (ELISA)

Wound tissues were homogenized, and the TNF α, IL 6, and IL 10 levels were measured using the enzyme-linked immunosorbent assay (ELISA) kits (TNF α, ab236712; IL 6, ab234570; IL 10, ab214566; Abcam, UK). Optical density (OD) was recorded at 450 nm, and cytokine concentrations were calculated from standard curves.

### Fe^2+^ detection

Tissue Fe^2+^ concentrations were determined using a colorimetric iron assay kit (MAK025, Sigma-Aldrich). Tissue samples were homogenized in 1 mL of distilled water and centrifuged to obtain the supernatant. Cell samples were processed by direct centrifugation. For each measurement, 100 µL of the supernatant was added to a flat-bottomed 96-well UV plate. A reaction mixture consisting of 35 µL reagent A, 5 µL reagent B, and 150 µL reagent C was added to each well and incubated for 5 min at ambient temperature. The absorbance was recorded at 359 nm using an Epoch microplate spectrophotometer. Fe^2+^ concentrations were normalized to protein levels. All experiments were performed in triplicate.

### Biochemical assays

Reduced glutathione (GSH) and oxidized glutathione (GSSG) levels in HaCaT cells and wound tissues were measured using a GSH/GSSG assay kit (S0053, Beyotime). The absorbance was recorded at 450 nm using a microplate reader (E8051, Promega), and concentrations were calculated using standard curves. Malondialdehyde (MDA) content, a biomarker for lipid peroxidation, was quantified using an MDA assay kit (BC0025, Solarbio).

### Transmission electron microscopy (TEM) for mitochondrial morphology observation

Wound tissue samples were cut into 1-mm^3^ blocks immediately after collection and fixed in 2.5% glutaraldehyde (340855, Sigma-Aldrich, USA) at 4 °C for 24 h. Samples were washed three times in 0.1 M PBS (pH 7.4) for 15 min each, followed by fixation in 1% osmium tetroxide (20816-12-0, Sigma-Aldrich, USA) for 1 h and washing thrice in PBS. Dehydration was carried out through a graded ethanol series (30%, 50%, 70%, 90%, and 100%), and tissues were embedded in epoxy resin (02660R-AB, SPI Supplies, USA). Ultrathin sections (70 nm) were prepared using an ultramicrotome (Leica UC7, Germany), stained with uranyl acetate (2%) and lead citrate (1%), and then were examined using a transmission electron microscope (TEM) (Tecnai G2 Spirit BioTWIN, FEI, USA). Mitochondrial volume density, crista integrity, and number were quantified using ImageJ software (v1.53t). Volume density was calculated as the percentage of the cytoplasmic area occupied by the mitochondria. The cytoplasmic region was selected using the rectangle tool, and individual mitochondria were outlined using the wand tool. Normal and swollen mitochondria were distinguished by cristae preservation, morphology, electron density, and outer membrane continuity. The volume density was calculated as follows: volume density = (total mitochondrial area/cytoplasmic area) × 100%. Crista integrity was scored semi-quantitatively based on the continuity, organization, and evidence of vacuolization, fragmentation, or dissolution: 0 = normal; 1 = mild reduction; 2 = moderate reduction; 3 = severe reduction/loss. Crista density was assessed by counting the number of intersections between mitochondrial crista membranes and a standardized line drawn across each mitochondrial profile using the straight-line tool.

### Mitochondrial membrane potential (ΔΨm) assay (JC-1 staining)

Skin fibroblasts were isolated from wound tissue by trypsin digestion, seeded into 6-well plates (2 × 10^5^ cells/well) and cultured for 24 h ΔΨm was assessed using the JC-1 dye (C2006, Beyotime, China). Cells were incubated in 5 µM JC-1 working solution at 37 °C in the dark for 20 min and then washed twice in PBS. Fluorescence intensities were measured using a microplate reader (E8051, Promega, USA) at excitation/emission wavelengths of 525/590 nm for red and 485/530 nm for green. The red/green fluorescence ratio was calculated to evaluate ΔΨm, with higher ratios indicating enhanced membrane integrity.

### Adenosine triphosphate (ATP) content quantification

Wound tissue was flash-frozen in liquid nitrogen, and adenosine triphosphate (ATP) levels were determined using an ATP assay kit (S0026, Beyotime, China). A 50-mg tissue sample was homogenized in 0.5 mL ATP extraction buffer, followed by centrifugation at 12,000 rpm for 10 min at 4 °C, and the supernatant was extracted. A standard curve was generated using known ATP concentrations. After reagent mixing, the absorbance was measured at 570 nm, and ATP levels were normalized to total protein concentration (nmol/mg protein).

### Statistical analysis

All experiments were performed in triplicate or more. Data are expressed as mean ± standard deviation. Independent sample *t*-tests were used for between-group comparisons. One-way analysis of variance (ANOVA) was applied for multi-group comparisons, followed by Tukey’s honestly significant difference *post hoc* testing when appropriate. For data that did not meet assumptions of normality or homogeneity of variance, the Mann–Whitney U test or Kruskal–Wallis H test was used. Statistical analyses were carried out using GraphPad Prism 9.5.0 (GraphPad Software, Inc.) and R version 4.2.1 (R Foundation for Statistical Computing). A two-tailed *p*-value <0.05 was considered statistically significant; *p* ≥ 0.05 was regarded as nonsignificant.

## Results

### Transcriptomic Analysis Reveals that Rb1 promotes PI repair by inhibiting ferroptosis *via* the SIRT1–AMPK pathway

**FIGURE 1:**
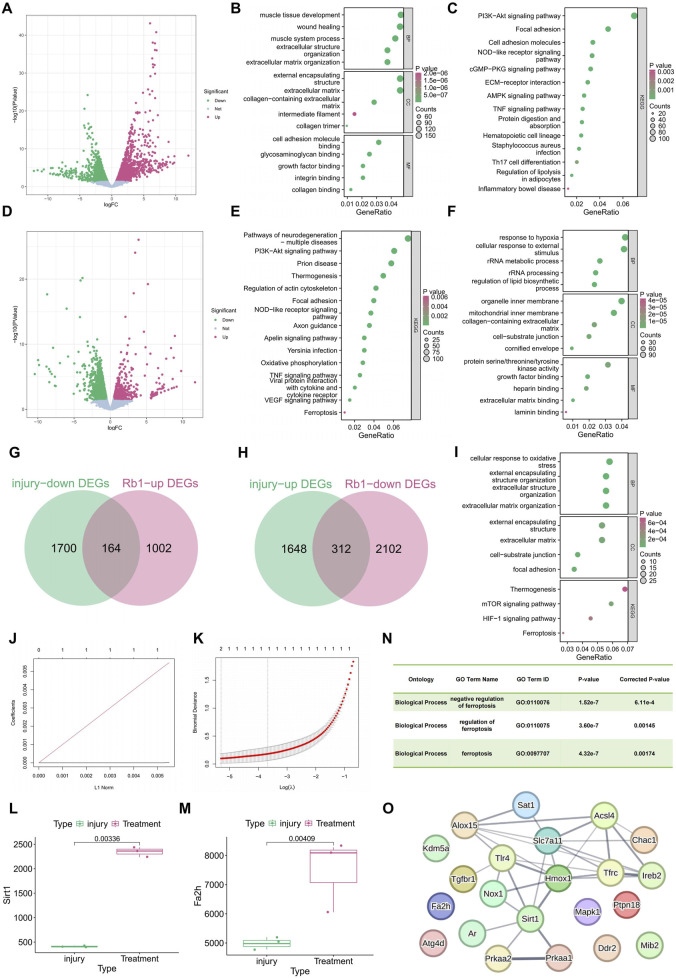
Transcriptomic analysis of the regulatory effects of Rb1 on pressure-injured skin. **(A)** Volcano plot showing DEGs between normal skin and pressure-injured skin (|log_2_fold change| > 0.5 and *p* < 0.05); **(B)** GO enrichment analysis of injury-related DEGs in terms of biological processes, cellular components, and molecular functions; **(C)** KEGG pathway enrichment analysis of injury-related DEGs; **(D)** volcano plot showing DEGs between pressure-injured skin and Rb1-treated skin (|log_2_fold change| > 0.5 and *p* < 0.05); **(E)** GO enrichment analysis of Rb1-related DEGs; **(F)** KEGG pathway enrichment analysis of Rb1-related DEGs; **(G)** Venn diagram showing the intersection between injury-DEGs and Rb1-DEGs; **(H)** Venn diagram showing the number of DEGs with reversed expression patterns; **(I)** GO and KEGG enrichment analyses of the 476 reversed DEGs; **(J)** LASSO regression analysis of the 476 reversed-expression DEGs with optimal lambda determined *via* cross-validation; **(K)** feature genes selected by LASSO regression; **(L,M)** qPCR validation of the expression levels of feature genes Fa2h and Sirt1; **(N)** GO and KEGG enrichment results of Rb1 analyzed using the CTD; **(O)** PPI network of Fa2h and Sirt1 with ferroptosis-related genes analyzed *via* the STRING database.

To elucidate the molecular mechanisms underlying PI, we first performed transcriptome sequencing on skin samples obtained from normal rats (Normal, n = 3) and PI rats (Injury, n = 3). Differential expression analysis identified 1,864 downregulated and 1,960 upregulated genes (|log_2_fold change| > 0.5 and *p* < 0.05), collectively referred to as injury-DEGs (Figure 1A). GO enrichment analysis showed that these DEGs were mainly involved in muscle tissue development, wound healing, and extracellular matrix (ECM) organization. Cellular component enrichment was observed in collagen fibers, intermediate filaments, and extracellular structures. Molecular function analysis highlighted associations with integrin binding, collagen binding, and cell adhesion molecule activity (Figure 1B). KEGG pathway analysis further revealed significant enrichment in PI3K–Akt signaling, cell adhesion molecules, NOD-like receptor signaling, and ECM–receptor interaction, reflecting the strong activation of inflammatory responses, ECM remodeling, and signal transduction in skin affected by PIs (Figure 1C). These findings indicate that PI induces a coordinated immune and reparative response, providing a foundation for mechanistic investigation and therapeutic targeting.

To investigate the therapeutic effects of Rb1, we next conducted transcriptome sequencing on skin tissues obtained from Rb1-treated PI rats (Treatment, n = 3) and compared with the results with those of untreated PI tissues. A total of 2,414 downregulated genes and 1,166 upregulated genes were identified as Rb1-DEGs (|log_2_fold change| > 0.5 and *p* < 0.05) (Figure 1D). GO analysis demonstrated enrichment in BP related to hypoxia response, lipid biosynthesis regulation, and rRNA metabolic processes. These genes were predominantly localized to mitochondrial inner membranes, ECM components, and cell junction regions. Molecular function analysis indicated involvement in ECM binding, laminin interaction, tyrosine kinase activity, and growth factor binding (Figure 1E). KEGG pathway enrichment showed that Rb1 treatment influenced several key pathways, including PI3K–Akt, NOD-like receptor, TNF signaling, oxidative phosphorylation, VEGF signaling, cytoskeletal regulation, ferroptosis, cytokine–receptor interactions, and neurodegeneration-related pathways (Figure 1F). These results imply that Rb1 facilitates PI repair by modulating energy metabolism, inflammation, cell survival, and ECM remodeling.

To identify key genes altered by PI and reversed by Rb1 treatment, upregulated and downregulated injury-DEGs (Normal vs. Injury) were intersected with downregulated and upregulated Rb1-DEGs (Injury vs. Treatment), respectively. As shown in Figures 1G,H, 476 genes exhibited reverse expression patterns in the two comparisons. Among them, the expressions of 164 genes were downregulated after injury but restored upon Rb1 treatment, while the expressions of 312 genes were upregulated after injury but suppressed following Rb1 intervention. These “expression-reversed genes” are considered potential functional mediators of Rb1-induced wound repair. GO enrichment of these reverse-expression genes showed significant involvement in oxidative stress responses, ECM remodeling, and cell adhesion. These genes were localized to extracellular envelopes, ECM regions, and cell–matrix junctions. Molecular functions were primarily associated with interactions between cells and the extracellular environment (Figure 1I). KEGG analysis further demonstrated marked enrichment in mTOR and HIF-1 signaling, together with ferroptosis, highlighting the roles of Rb1 in metabolic regulation, stress adaptation, and cell fate control (Figure 1I). These findings collectively suggest that Rb1 exerts multifaceted regulatory effects on PI repair at the system level.

To further indicate therapeutic targets, we applied LASSO regression modeling to the 476 reverse-expression DEGs and identified the optimal lambda *via* cross-validation (Figure 1J). Two genes with non-zero coefficients, Fa2h and Sirt1, were selected, the expressions of which were significantly upregulated following Rb1 treatment (Figures 1K–M). Analysis using the CTD revealed ferroptosis as a major pathway targeted by Rb1 (Figure 1N). Among the 476 reverse-expression DEGs, 21 were associated with ferroptosis, including Sirt1. PPI network analysis showed that Sirt1 is closely linked to multiple ferroptosis regulators and directly interacts with AMPK-encoding genes Prkaa1 and Prkaa2 (Figure 1O), suggesting that Sirt1 may serve as a key mediator of Rb1’s therapeutic effects.

The collective evidence supports a mechanism in which Rb1 promotes tissue repair in PI by activating the SIRT1–AMPK signaling axis and suppressing ferroptosis. The observations provide a mechanistic basis for subsequent cellular and animal studies designed to define Rb1-mediated protection in greater detail.

### Rb1 activates the SIRT1–AMPK pathway to inhibit ferroptosis and enhance cell viability

**FIGURE 2:**
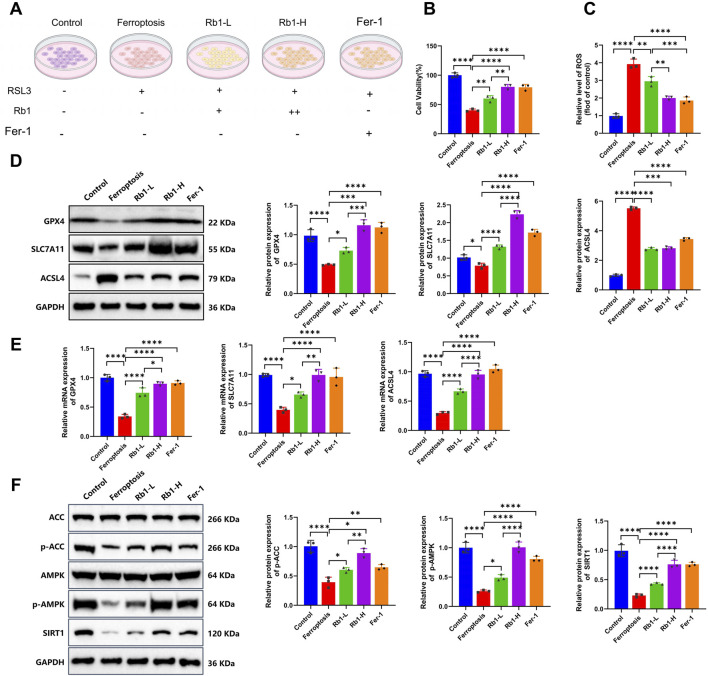
Validation of the role of Rb1 in regulating the SIRT1–AMPK pathway and ferroptosis. **(A)** Schematic diagram of experimental groups: “RSL3+” indicates treatment with 2 μM RSL3; “Rb1+” indicates 10 μM Rb1; “Rb1++” indicates 20 μM Rb1; “Fer-1+” indicates 2 μM Fer-1; **(B)** cell viability assessed using the CCK-8 assay; **(C)** intracellular ROS levels; **(D,E)** Western blot and RT-qPCR analysis of ferroptosis-related molecules (GPX4, SLC7A11, and ACSL4). Relative protein expression = target protein/GAPDH, normalized to the control group (set to 1.0); **(F)** Western blot analysis of SIRT1–AMPK pathway proteins (SIRT1, AMPK, p-AMPK, ACC, and p-ACC). Relative protein expression calculated as SIRT1/GAPDH, p-AMPK/AMPK, and p-ACC/ACC, normalized to the control group. All data represent at least three independent experiments and are expressed as mean ± SD. Two-group comparisons were analyzed using an independent-sample t-test. *p < 0.05, **p < 0.01, and ***p < 0.001.

To investigate the role of Rb1 in ferroptosis and the SIRT1–AMPK signaling pathway, we conducted a series of *in vitro* cellular experiments. Experimental groups included Control (untreated), Ferroptosis (ferroptosis-induced), Rb1-L (ferroptosis-induced with low-dose Rb1), and Rb1-H (ferroptosis-induced with high-dose Rb1), along with the Fer-1 group (ferroptosis positive-control group treated with the ferroptosis inhibitor Fer-1 and high-dose Rb1) (Figure 2A). Cell viability was evaluated using the CCK-8 assay. The Ferroptosis group showed a marked reduction in cell viability compared with the Control group, confirming successful induction of ferroptosis. Both Rb1-treated groups exhibited marked restoration of cell viability, with the Rb1-H group demonstrating an effect similar to that of the Fer-1 group (Figure 2B). Intracellular ROS levels were measured using the DCFH-DA fluorescent probe. The Ferroptosis group displayed substantial ROS accumulation, whereas Rb1 treatment markedly reduced ROS levels. ROS levels in the Rb1-H group approached those observed in the Control and Fer-1 groups, indicating that Rb1 effectively suppresses oxidative stress associated with ferroptosis (Figure 2C).

Western blot and RT-qPCR analyses were performed to examine the expressions of ferroptosis-related molecules. Compared with the Control group, the Ferroptosis group showed marked reductions in GPX4 and SLC7A11 expressions at both protein and mRNA levels, together with increased ACSL4 expression, indicating disruption of the antioxidant defense system. Rb1 treatment reversed these alterations in a dose-dependent manner, and the Rb1-H group exhibited effects similar to those in the Fer-1 group, suggesting that Rb1 suppresses ferroptosis by restoring redox homeostasis (Figures 2D,E). We further evaluated the activation status of the SIRT1–AMPK signaling axis. Protein expression levels of SIRT1, p-AMPK, and p-ACC were markedly reduced in the Ferroptosis group, indicating suppression of energy metabolic activity. Rb1 administration restored the expression of these proteins, and high-dose treatment produced levels compared to those in the Control and Fer-1 groups. These findings indicate that Rb1 reactivates the SIRT1–AMPK axis and promotes metabolic recovery under ferroptotic stress (Figure 2F).

Collectively, our results demonstrate that Rb1 attenuates ferroptosis and promotes cell survival by activating the SIRT1-dependent AMPK signaling pathway, providing a potential molecular mechanism for PI wound regeneration.

### Rb1 inhibits ferroptosis *via* activation of the SIRT1–AMPK pathway to promote cell survival

**FIGURE 3:**
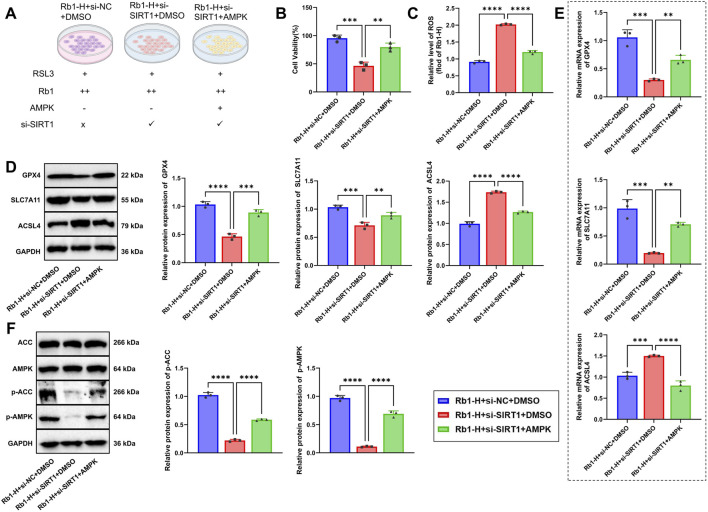
*In vitro* evidence that Rb1 suppresses ferroptosis *via* the SIRT1–AMPK pathway. **(A)** Schematic diagram of experimental groups. “RSL3+” indicates treatment with 2 μM RSL3; “Rb1+” indicates 10 μM Rb1; “Rb1++” indicates 20 μM Rb1; “AMPK+” indicates the 0.5 mM AMPK activator; **(B)** cell viability determined using the CCK-8 assay; **(C)** intracellular ROS levels measured using the DCFH-DA fluorescent probe; **(D,E)** Western blot and RT-qPCR analyses of ferroptosis-related molecules GPX4, SLC7A11, and ACSL4 at the protein and mRNA levels. Relative expression levels of GPX4, SLC7A11, and ACSL4 were calculated as the ratio of the target protein gray value to that of GAPDH. The Rb1-H + si-NC + DMSO group was set as the baseline (1.0), and the relative expression levels of other groups were normalized accordingly; **(F)** Western blot analysis of SIRT1–AMPK pathway-related proteins, including p-AMPK, ACC, and p-ACC. Relative p-AMPK expression was calculated as the ratio of p-AMPK to AMPK, and relative p-ACC expression was calculated as the ratio of p-ACC to ACC. The Rb1-H + si-NC + DMSO group was set as the baseline (1.0), and other groups were normalized to this reference. All data are presented as mean ± standard deviation (Mean ± SD) from at least three independent experiments. Comparisons between two groups were performed using an independent-sample t-test. *p < 0.05, **p < 0.01, and ***p < 0.001.

We investigated the mechanism by which Rb1 inhibits ferroptosis through activation of the SIRT1–AMPK signaling axis. Experimental groups included the Rb1-H + si-NC + DMSO group (ferroptosis induction + high-dose Rb1 + siRNA-negative control + AMPK-activator control), the Rb1-H + si-SIRT1 + DMSO group (ferroptosis + high-dose Rb1 + SIRT1 knockdown + AMPK-activator control), and the Rb1-H + si-SIRT1 + AMPK group (ferroptosis induction + high-dose Rb1 + SIRT1 knockdown + AMPK activator) (Figure 3A). Among the two siRNAs tested, si-SIRT1-2 demonstrated more effective silencing of SIRT1 compared to si-SIRT1-1. Therefore, si-SIRT1-2 was used in subsequent experiments and designated as si-SIRT1 (Supplementary Figure S1A,B). Cell viability was assessed using the CCK-8 assay. Compared with the Rb1-H + si-NC + DMSO group, the cell viability was significantly decreased in the Rb1-H + si-SIRT1+DMSO group, indicating that SIRT1 knockdown partially reversed the protective effects of Rb1 against ferroptosis. Notably, the addition of an AMPK activator in the Rb1-H + si-SIRT1 + AMPK group partially restored cell viability, suggesting that AMPK activation can compensate for the loss of SIRT1 function (Figure 3B). Consistent with this, intracellular ROS levels measured using the DCFH-DA probe were markedly elevated in the Rb1-H + si-SIRT1 + DMSO group, whereas AMPK activation significantly reduced ROS accumulation in the Rb1-H + si-SIRT1 + AMPK group (Figure 3C).

Western blot and RT-qPCR analyses revealed that knockdown of SIRT1 in the Rb1-H + si-NC + DMSO group markedly reduced GPX4 and SLC7A11 expressions at both mRNA and protein levels while increasing ACSL4 expression. These alterations were reversed in the Rb1-H + si-SIRT1 + DMSO group, indicating that AMPK activation restores antioxidant defense mechanisms (Figures 3D,E). Furthermore, we assessed the activation status of the SIRT1–AMPK pathway. In the Rb1-H + si-SIRT1 + DMSO group, phosphorylation levels of AMPK and its downstream target ACC were substantially reduced, whereas AMPK activation restored phosphorylation levels of both proteins in the Rb1-H + si-SIRT1 + AMPK group, demonstrating that AMPK activators can bypass SIRT1 to directly stimulate the AMPK pathway (Figure 3F).

Collectively, the findings indicate that Rb1 suppresses ferroptosis through activation of the SIRT1-dependent AMPK signaling cascade. Pharmacological activation of AMPK partially compensates for SIRT1 deficiency, supporting the central role of the SIRT1–AMPK axis in regulating ferroptotic cell death.

### Rb1 promotes PI wound healing and inhibits ferroptosis

**FIGURE 4:**
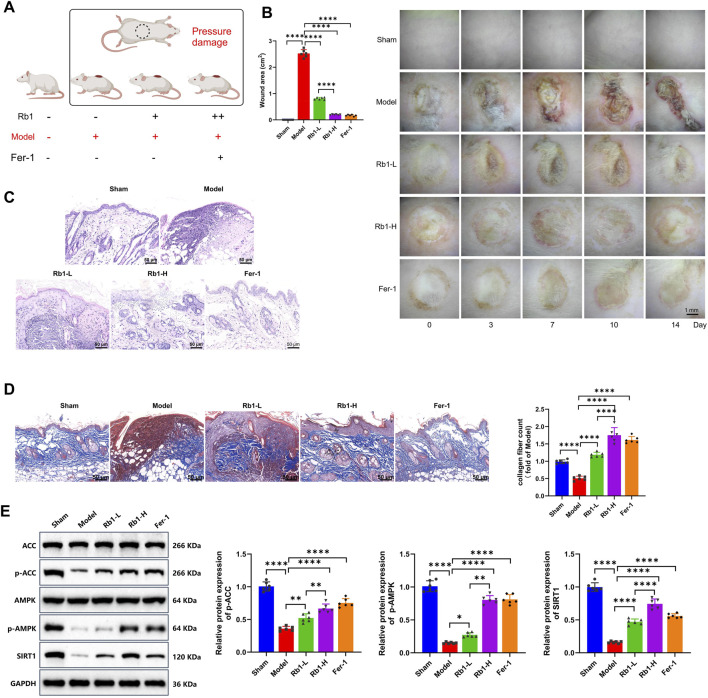
*In vivo* validation of Rb1 in promoting PI wound healing and inhibiting ferroptosis. **(A)** Schematic diagram of the experimental groups. “Rb1+” indicates treatment with 2 mg/kg Rb1; “Rb1++” indicates 10 mg/kg Rb1; “Fer-1+” indicates 10 mg/kg Fer-1; **(B)** evaluation of wound healing by measuring the wound area. Representative images are shown on the left, and the quantified wound area on day 14 is shown on the right. Scale bar: 1 mm; **(C)** H&E staining showing histopathological changes in wound tissues. Scale bar: 50 μm. **(D)** Masson’s staining showing histopathological changes in wound tissues. Scale bar: 50 μm. **(E)** Western blot analysis of SIRT1–AMPK pathway-related proteins, including SIRT1, AMPK, p-AMPK, ACC, and p-ACC. Relative expression levels were calculated as follows: SIRT1 relative expression = SIRT1 protein gray value/GAPDH gray value; p-AMPK relative expression = p-AMPK/AMPK; p-ACC relative expression = p-ACC/ACC. The sham group was set as the baseline value (1.0), and the relative expression levels of the other groups were normalized accordingly. Each group contained six animals. All data are presented as mean ± standard deviation (Mean ± SD) from at least three independent experiments. Comparisons between two groups were performed using an independent-sample t-test. *p < 0.05, **p < 0.01, and ***p < 0.001.

To investigate the role of Rb1 in promoting wound regeneration following PI, a PI model was established in SD rats. The animals were divided into five groups: Sham, Model, Rb1-L (low-dose, 2 mg/kg), Rb1-H (high-dose, 10 mg/kg), and Fer-1 (Figure 4A). Serial imaging and wound area quantification showed delayed wound healing in the Model group compared with the Sham group. Both Fer-1-and Rb1-treated groups demonstrated accelerated wound closure, and the Rb1-H group exhibited the most pronounced improvement (Figure 4B; Supplementary Table S3). Histopathological analysis further confirmed these findings. The Model group displayed pronounced inflammatory cell infiltration and reduced granulation tissue and collagen fiber formation. Treatment with Fer-1 and Rb1 notably reversed these pathological alterations, particularly in the high-dose group (Figures 4C,D).

Western blot analysis revealed marked reductions in SIRT1, p-AMPK, and p-ACC expressions in the Model group. Treatment with Fer-1 and Rb1 restored the expressions of these proteins, and the Rb1-H group displayed the highest expression levels (Figure 4E).

These findings suggest that Rb1 enhances wound regeneration in PI by inhibiting ferroptosis *via* activation of the SIRT1–AMPK signaling pathway, with a dose-dependent therapeutic effect.

### Rb1 attenuates oxidative stress and inflammatory responses in PI

**FIGURE 5:**
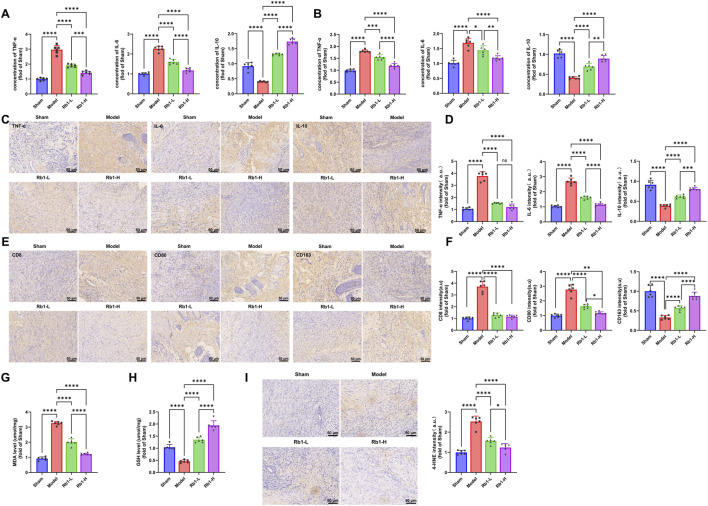
Rb1 attenuates oxidative stress and suppresses inflammation in PI. **(A)** ELISA quantification of serum TNF-α, IL-6, and IL-10 levels; **(B)** ELISA detection of TNF-α, IL-6, and IL-10 levels in homogenates of dorsal wound tissues; **(C)** immunohistochemical analysis of TNF-α, IL-6, and IL-10 expressions in dorsal wound tissues (bar: 25 μm); **(D)** quantitative analysis of inflammatory cytokines across groups; **(E)** immunohistochemical analysis of CD8, CD80, and CD163 expressions in dorsal wound tissues. Scale bar: 25 μm; **(F)** quantification of CD8, CD80, and CD163 expressions across different treatment groups; **(G)** quantification of MDA levels in different treatment groups; **(H)** quantification of GSH levels in different treatment groups; **(I)** immunohistochemical analysis of 4-HNE expression in dorsal wound tissues (Scale bar: 25 μm). Each group contained six animals. All data are presented as mean ± standard deviation (Mean ± SD). Comparisons between two groups were performed using an independent-sample t-test. *p < 0.05, **p < 0.01, and ***p < 0.001 compared between groups.

The anti-inflammatory effects of Rb1 in PI were further examined using the same animal model and grouping strategy. ELISA results showed that the Model group exhibited significantly elevated serum levels of TNF-α and IL-6 compared with the Sham group (Figure 5A). Similar trends were observed in wound tissue homogenates, accompanied by reduced IL-10 levels (Figure 5B). Rb1 treatment significantly decreased TNF-α and IL-6 concentrations and increased IL-10 levels, and the Rb1-H group demonstrated the most pronounced improvement.

Immunohistochemical analysis supported these findings. TNF-α and IL-6 expressions were markedly elevated in wound tissues of the Model group, whereas IL-10 expression was reduced. High-dose Rb1 administration reversed these alterations and shifted cytokine expression toward an anti-inflammatory profile (Figures 5C,D). The immune cell distribution was also assessed. The Model group showed increased CD8 and CD80 expressions and reduced CD163 expression, indicating enhanced infiltration of CD8^+^ T-cell and M1 macrophage infiltration and diminished M2 macrophage polarization. Rb1-L and Rb1-H treatment reduced CD8 and CD80 expressions and increased CD163 expression levels, indicating reduced CD8^+^ T-cell and M1 macrophage infiltration and enhanced M2 macrophage polarization (Figures 5E,F).

Oxidative stress markers were subsequently evaluated. MDA levels were significantly increased, and GSH levels were decreased in both serum and tissue samples from the Model group. Rb1 treatment reversed these changes, and the Rb1-H group exhibited the strongest antioxidant effect (Figures 5G,H).

Immunofluorescence staining further showed elevated 4-HNE expression in the Model group, whereas both Rb1-L and Rb1-H markedly suppressed 4-HNE levels, indicating substantial inhibition of lipid peroxidation (Figure 5I).

Collectively, these findings indicate that Rb1 alleviates oxidative stress and ferroptosis, thereby enhancing wound repair in PI.

### Rb1 Enhances Mitochondrial Function, Reduces Oxidative Stress, and inhibits ferroptosis

**FIGURE 6:**
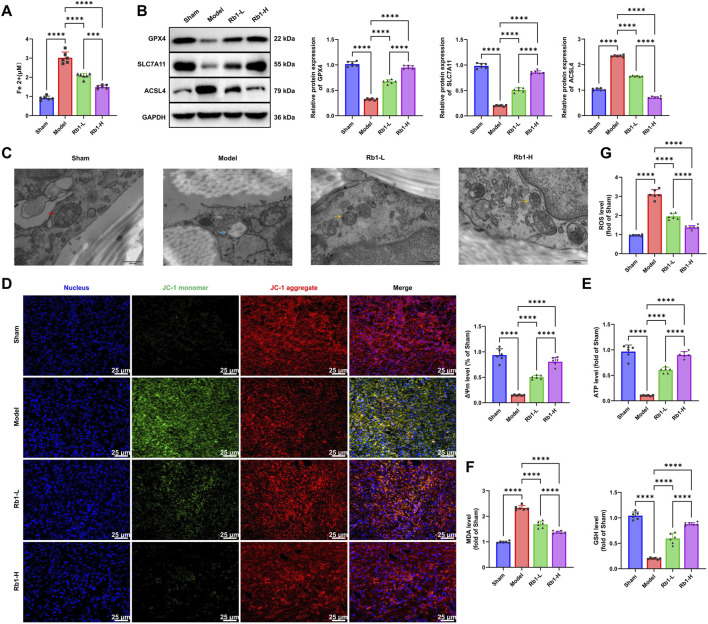
Rb1 enhances mitochondrial function, reduces oxidative stress, and inhibits ferroptosis. **(A)** Measurement of Fe^2+^ concentration in wound tissues; **(B)** detection of ferroptosis-related proteins ACSL4, SLC7A11, and GPX4 in wound tissues. Relative expression levels of GPX4, SLC7A11, and ACSL4 were calculated as the ratio of the target protein gray value to that of GAPDH. The sham group was set as the baseline (1.0), and the relative expression levels of other groups were normalized accordingly; **(C)** TEM of mitochondrial morphology in wound tissues—red arrows indicate normal mitochondria, blue arrows indicate swollen mitochondria, and yellow arrows indicate restored mitochondria (scale bar: 500 nm); **(D)** JC-1 staining for assessing changes in mitochondrial membrane potential (ΔΨm) in wound tissues of each group. Scale bar: 25 μm; **(E)** Quantitative analysis of ATP levels in wound tissues from each group; **(F)** measurement of GSH and MDA levels to assess oxidative stress in wound tissues from each group; **(G)** immunofluorescence detection of ROS levels in wound tissues from each group. Each group contained six animals. All data are presented as mean ± standard deviation (Mean ± SD). Comparisons between two groups were performed using an independent-sample t-test. *p < 0.05, **p < 0.01, and ***p < 0.001.

To further investigate the effects of Rb1 on ferroptosis and oxidative stress in PI, we measured Fe^2+^ concentrations and ferroptosis-related protein expression in wound tissues. The Model group showed significantly elevated Fe^2+^ levels and increased ACSL4 expression compared with the Sham group, along with marked reductions in SLC7A11 and GPX4 expressions. Rb1 treatment reduced Fe^2+^ levels and ACSL4 expression in a dose-dependent manner, and the Rb1-H group exhibited the strongest effect. SLC7A11 and GPX4 expressions increased substantially in both Rb1-treated groups, with the greatest upregulation observed in Rb1-H (Figures 6A,B).

Subsequently, we examined the mitochondrial ultrastructure using TEM and assessed ΔΨm *via* JC-1 staining. TEM images demonstrated that the mitochondria in the Model group were swollen, with disrupted cristae and compromised membrane structure (Figure 6C). Both the Rb1-L and Rb1-H groups displayed improved mitochondrial morphology, characterized by reduced swelling and restored cristae architecture, with more substantial recovery observed in Rb1-H (Figure 6C). JC-1 staining further demonstrated a significant decrease in ΔΨm in the Model group, indicating mitochondrial dysfunction. In comparison, both Rb1-treated groups showed substantial restoration of ΔΨm, with the Rb1-H group exhibiting the most pronounced improvement (Figure 6D). These findings suggest that Rb1 preserves mitochondrial structural integrity and stabilizes membrane potential, which may underlie its capacity to mitigate the effects of oxidative stress and inhibit ferroptosis.

To examine the regulatory effects of Rb1 on energy metabolism, ATP levels were measured in wound tissues together with oxidative stress markers MDA and GSH. ATP levels were markedly reduced in the Model group, indicating impaired energy metabolism. Rb1 treatment significantly increased ATP production, and the Rb1-H group showed the greatest enhancement (Figure 6E).

Additionally, oxidative stress assessments showed elevated MDA and decreased GSH levels in the Model group, indicating enhanced lipid peroxidation (Figure 6F). Immunofluorescence staining revealed that ROS levels were significantly increased in the Model group, whereas both Rb1-L and Rb1-H groups exhibited notable reductions in ROS accumulation, with the high-dose group showing pronounced improvement (Figure 6G).

In summary, these results demonstrate that Rb1 inhibits ferroptosis by preserving mitochondrial integrity, stabilizing membrane potential, promoting ATP synthesis, and attenuating oxidative stress. This underscores the therapeutic potential of Rb1 in the treatment of PI and highlights its mitochondria-protective mechanisms.

## Discussion

This study is the first to systematically elucidate the pivotal regulatory role of the SIRT1–AMPK signaling axis in the repair of PI wounds, addressing a critical gap in the current literature. Although previous research has predominantly focused on the anti-apoptotic and antioxidant functions of the SIRT1/AMPK pathway in metabolic disorders, cardiovascular and cerebrovascular diseases, and ischemic injuries, its mechanistic involvement in chronic skin wounds, particularly PI, remains insufficiently explored (Valente et al., 2016; Zheng J. et al., 2021). Through integrated transcriptomic profiling and functional validation, the study identified significant activation of SIRT1 and its downstream target p-AMPK following Rb1 treatment, indicating that this pathway acts as a key regulatory node in PI tissue. Moreover, *in vivo* and *in vitro* experiments confirmed that inhibition of either SIRT1 or AMPK activity markedly reduced the protective effects of Rb1 against ferroptosis, reinforcing the essential role of this signaling axis in wound repair. These findings provide a new perspective on the molecular networks involved in PI regeneration. In contrast to studies focused on single molecular targets, our multi-layered investigation of the entire signaling axis provides a more robust biological rationale. Moreover, activation of the SIRT1–AMPK pathway may influence additional biological processes, including energy metabolism, cell migration, and inflammatory modulation, thereby expanding potential directions for future research.

Ferroptosis, a recently recognized form of programmed cell death closely associated with inflammation and oxidative stress, has been extensively studied in cancer, neurodegenerative conditions, and metabolic disorders (Wahida and Conrad, 2023). Existing research indicates that ferroptosis contributes to cellular dysfunction and tissue necrosis in chronic wounds such as diabetic foot ulcers and burns. However, its precise role in PI remains poorly defined, with relatively few studies addressing this question (Han et al., 2023). In this study, we used erastin- and RSL3-induced ferroptosis models to replicate lipid peroxidation and ROS accumulation characteristic of the PI microenvironment, and systematically assessed the protective effects of Rb1 under these conditions. The results demonstrate that Rb1 prevents the downregulation of expressions of key antioxidant molecules, including GPX4 and SLC7A11, thereby maintaining redox balance and reducing ROS-mediated injury. Expression of the ferroptosis-associated protein ACSL4 was also significantly downregulated following Rb1 treatment, suggesting that Rb1 may modulate ferroptosis through regulation of lipid metabolic processes. In contrast to previous PI studies that have largely focused on inflammatory pathways, our findings provide the first cell-based evidence implicating ferroptosis in the persistence of PI, establishing a new conceptual framework for understanding its pathological progression.

Rb1, a representative bioactive constituent of the traditional Chinese medicinal herb *Panax ginseng*, has garnered increasing attention for its antioxidant, anti-inflammatory, and neuroprotective properties (Gao et al., 2020; Shaukat et al., 2020). Pharmacological studies have shown that Rb1 exerts multi-target cytoprotective actions by activating several signaling pathways, including Nrf2, PI3K/Akt, and ERK (Zhou et al., 2024; Chen X. et al., 2019). Most existing research works have focused on neurological disorders, cardiovascular diseases, and diabetes-related complications (Qin et al., 2019; Zhou et al., 2019), whereas its therapeutic potential in PI has not been investigated. The capacity of Rb1 to inhibit ferroptosis through the SIRT1/AMPK pathway, modulate the wound microenvironment, and promote tissue regeneration has not been systematically examined (Wang et al., 2021; Qiang et al., 2017). A key innovation of this study lies in its integration of active constituents from TCM with multi-omics strategies, thereby addressing the long-standing challenge of lack of mechanistic insights in herbal medicine research. By clarifying the causal relationship between Rb1 and its downstream molecular targets, the study expands the therapeutic scope of Rb1 and establishes a reproducible, evidence-based framework that supports the modernization of traditional medicine. The findings provide both mechanistic insights and practical translational values.

The study also demonstrated that ginsenoside Rb1 reduced inflammatory cytokine levels within wound tissue and in serum. Local inflammatory responses are rapidly initiated following tissue injury to remove harmful stimuli and promote repair. Severe injury or uncontrolled infection can lead to excessive production of inflammatory mediators that enter the circulatory system and induce systemic inflammatory responses. Local inflammation acts as the origin, whereas systemic inflammation emerges when locally produced mediators enter the bloodstream, forming a continuous and unified pathological process (Lord et al., 2014; Klyne et al., 2021). The present findings suggest that ginsenoside Rb1 alleviates local inflammation to support wound healing and may concurrently attenuate systemic inflammation secondary to local inflammatory activation, thereby reducing the overall symptom burden.

To elucidate the underlying mechanisms, RNA-seq analysis was conducted on skin tissue samples across experimental groups. GO and KEGG enrichment analyses were used to systematically identify DEGs associated with wound healing. Integrated analyses incorporating network pharmacology and PPI network construction further refined the selection of key regulatory nodes, including SIRT1 and AMPK. Compared with traditional candidate gene-based strategies, the multi-omics methodology offers a more holistic and unbiased perspective, facilitating the discovery of novel targets from a systems biology standpoint. The application of network pharmacology highlights the inherently multi-target, multi-pathway nature of TCM, rendering the mechanisms of single compounds or complex formulas more systematic and quantifiable. Furthermore, this strategy provides a predictive framework for identifying potential synergistic interactions, laying the groundwork for future development of combination therapies.

To enhance the reliability and translational relevance of the findings, a dual experimental system combining an *in vitro* co-culture model with an *in vivo* PI model in SD rats was established. *In vitro*, a co-culture of L929 fibroblasts and HaCaT keratinocytes was used to more accurately simulate the skin microenvironment, thereby improving the physiological relevance. Ferroptosis was reliably induced using erastin and RSL3, providing a robust platform for evaluating the effects of Rb1 intervention. *In vivo*, PIs were generated in SD rats through sustained mechanical compression, closely mimicking clinical pathophysiological conditions. Rb1 treatment consistently activated the SIRT1/AMPK pathway, reduced ROS levels, and enhanced tissue repair in both cellular and animal models. The convergence of results across *in vitro* and *in vivo* systems strengthens the validity of the conclusions and underscores the therapeutic potential of Rb1 for clinical translation.

The microenvironment of PI wounds is characterized by chronic inflammation and elevated oxidative stress, two interrelated processes that reinforce each other and create a vicious cycle that severely impairs healing. Recent studies have shown that ferroptosis exacerbates this condition by enhancing ROS production and releasing proinflammatory cytokines such as TNF-α and IL-6, thereby amplifying local tissue damage. The present study shows that Rb1 reduces ROS levels through activation of the SIRT1/AMPK signaling axis and suppresses the expression of proinflammatory cytokines (e.g., TNF-α and IL-6) while upregulating those of the anti-inflammatory cytokine IL-10, indicating a robust immunomodulatory effect. These findings suggest that the SIRT1/AMPK pathway may serve as a central node in coordinating inflammatory and oxidative responses, representing a promising therapeutic target for PI management. In contrast to conventional wound healing studies that typically examine isolated pathological processes, the present work highlights the interplay among multiple mechanisms and provides a more integrated conceptual framework for addressing complex wound pathology.

Nevertheless, several limitations should be acknowledged. Although the study establishes the involvement of the SIRT1–AMPK pathway, downstream regulatory mechanisms and intercellular interactions influenced by Rb1 in ferroptosis were not fully delineated. Future studies incorporating single-cell transcriptomics and metabolomics may clarify cell-specific functions and broader systemic effects. Previous studies have reported that sex differences can influence wound healing (Ashcroft, 2004; Thomason et al., 2015). However, no significant differences between male and female rats were observed in our study. Consequently, all analyses were performed using pooled data from both sexes. This outcome may reflect the characteristics of the wound model or indicate that Rb1 lacks sex dependent variation in therapeutic efficacy. Additional investigations will be needed to address this question. Although *in vivo* experiments demonstrated therapeutic benefits, further validation in large animal models and clinical studies remains essential. Integration of Rb1 with advanced biomaterials or nanoscale delivery systems may improve tissue-specific targeting and enhance the translational potential. Examination of potential synergy between Rb1 and current wound therapies, including stem cell-based interventions or bioengineered dressings, also represents a promising direction for future research.

## Conclusion

This study systematically elucidates the mechanism by which Rb1 promotes the repair of PI wounds by activating the SIRT1–AMPK signaling cascade and suppressing ferroptosis. Transcriptomic profiling identified the SIRT1–AMPK axis and ferroptosis-related effectors, including GPX4 and SLC7A11, as key molecular targets of Rb1 intervention. *In vitro* experiments demonstrated that Rb1 markedly reduced ROS levels, inhibited erastin-/RSL3-induced ferroptosis, and enhanced SIRT1–AMPK pathway activation. Pharmacologic blockade of SIRT1 or AMPK reversed these protective effects, emphasizing the central regulatory role of this pathway. *In vivo* validation further confirmed that Rb1 accelerated wound closure, modulated the inflammatory milieu, improved mitochondrial function, and facilitated tissue regeneration by attenuating lipid peroxidation and ferroptotic cell death. Rescue experiments corroborated the critical regulatory function of SIRT1–AMPK signaling in ferroptosis, providing mechanistic support for precision therapeutic strategies targeting PI.

By integrating transcriptomic analysis, mechanistic investigation, and *in vitro* and *in vivo* validation, this study provides a comprehensive understanding of how Rb1 modulates ferroptosis and energy metabolism to enhance PI wound healing. The findings advance current concepts regarding the role of ferroptosis in chronic wound repair and support the therapeutic potential of bioactive constituents from traditional Chinese medicine in regenerative medicine. Future work may focus on optimizing delivery systems and developing targeted interventions aimed at ferroptosis and metabolic dysregulation, thereby expanding therapeutic opportunities for refractory wounds such as diabetic ulcers and PI.

## Data Availability

The original contributions presented in the study are publicly available. The RNA sequencing data have been deposited in the NCBI BioProject database under accession number PRJNA1417810.

## Glossary

ACC: Acetyl-CoA carboxylase
AICAR: 5-Aminoimidazole-4-carboxamide ribonucleotide
AMPK: AMP-activated protein kinase
ANOVA: Analysis of variance
ATP: Adenosine triphosphate
BCA: Bicinchoninic acid assay
BSA: Bovine serum albumin
CCK-8: Cell counting kit-8
DAB: Diaminobenzidine
DCFH-DA: 2′,7′-Dichlorofluorescin diacetate
DEGs: Differentially expressed genes
ΔΨm: Mitochondrial membrane potential
ECL: Enhanced chemiluminescence
ELISA: Enzyme-linked immunosorbent assay
FBS: Fetal bovine serum
4-HNE: 4-Hydroxynonenal
GO: Gene Ontology
GPX4: Glutathione peroxidase 4
GSH: Glutathione
GSSG: Glutathione disulfide
H&E: Hematoxylin and eosin
HRP: Horseradish peroxidase
IL-1β: Interleukin-1β
IL-6: Interleukin-6
JC-1: 5,5′,6,6′-Tetrachloro-1,1′,3,3′-tetraethylbenzimidazolylcarbocyanine iodide
KEGG: Kyoto Encyclopedia of Genes and Genomes
LASSO: Least absolute shrinkage and selection operator
MDA: Malondialdehyde
PBS: Phosphate-buffered saline
PI: Pressure injury
PPI: Protein–protein interaction
PVDF: Polyvinylidene fluoride
RFU: Relative fluorescence unit
RIPA: Radio-immunoprecipitation assay
RNA-seq: RNA sequencing
ROS: Reactive oxygen species
RT-qPCR: Real-time quantitative polymerase chain reaction
Rb1: Ginsenoside Rb1
SD: Sprague–Dawley
SDS-PAGE: Sodium dodecyl sulfate–polyacrylamide gel electrophoresis
siRNA: Small interfering RNA
SIRT1: Sirtuin 1
SLC7A11: Solute carrier family 7 member 11
TEM: Transmission electron microscopy
TNF-α: Tumor necrosis factor-α
