# Supplementary material for: Pharmacological activation of SIRT1–AMPK by ginsenoside Rb1: a novel therapeutic strategy for pressure injury via dual suppression of ferroptosis and inflammation
Source: Front Pharmacol. 2026 Feb 17;16:1683479. doi: 10.3389/fphar.2025.1683479 (PMC12953485; doi:10.3389/fphar.2025.1683479)
Supplement: Supplementary file 1 [file Table1.docx]

**Table S1. Sequence of RT-qPCR.**

| **Name** | **Sequence (5’-3’)** |
| --- | --- |
| GPX4 (human) | Forward: 5’- GAAGCAGGAGCCAGGGAGTA -3’ |
|  | Reverse: 5’- GGTGAAGTTCCACTTGATGGC -3’ |
| SLC7A11 (human) | Forward: 5’-TGCTGGGCTGATTTTATCTTCG-3’ |
|  | Reverse: 5’-GAAAGGGCAACCATGAAGAGG -3’ |
| ACSL4 (human) | Forward: 5’-TCTTCTCCGCTTACACTCTCT -3’ |
|  | Reverse: 5’-CTTATAAATTCTATCCATGATTTCCGGA -3’ |
| SIRT1 (human) | Forward: 5’-GCAACATCTTATGATTGGCACAG -3’ |
|  | Reverse: 5’-TTTGGATTCCCGCAACCTG -3’ |
| SIRT2 (human) | Forward: 5’-CTCTATCCTGGGCAGTTCAA -3’ |
|  | Reverse: 5’-CAGCTTAGCGGGTATTCGTG -3’ |
| GAPDH (human) | Forward: 5’- CGGAGTCAACGGATTTGGTCGTAT -3’ |
|  | Reverse: 5’- AGCCTTCTCCATGGTGGTGAAGAC -3’ |

Abbreviations: F, forward; R, reverse.
