# Supplementary figures and images for: Pharmacological activation of SIRT1–AMPK by ginsenoside Rb1: a novel therapeutic strategy for pressure injury via dual suppression of ferroptosis and inflammation
Source: Front Pharmacol. 2026 Feb 17;16:1683479. doi: 10.3389/fphar.2025.1683479 (PMC12953485; doi:10.3389/fphar.2025.1683479)

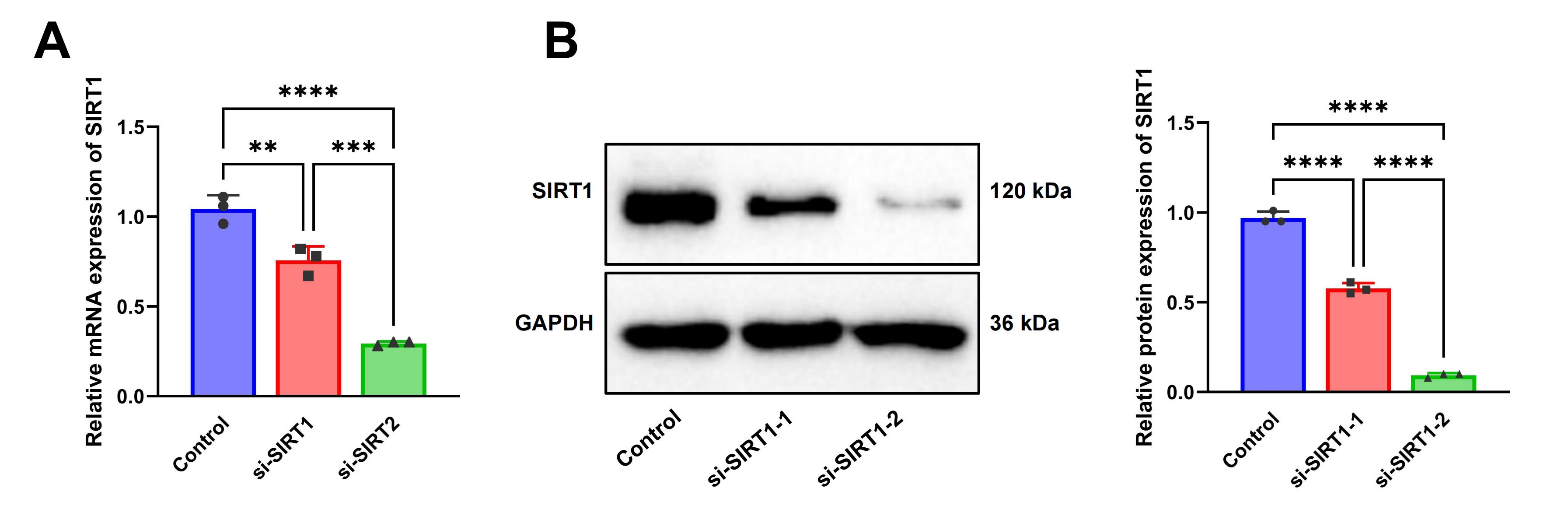

Supplement: Supplementary file 2 [file Image1.jpeg]
