# Supplementary material for: Pharmacological activation of SIRT1–AMPK by ginsenoside Rb1: a novel therapeutic strategy for pressure injury via dual suppression of ferroptosis and inflammation
Source: Front Pharmacol. 2026 Feb 17;16:1683479. doi: 10.3389/fphar.2025.1683479 (PMC12953485; doi:10.3389/fphar.2025.1683479)
Supplement: Supplementary file 3 [file Table2.docx]

**Table S2. Primary Antibody Information.**

| **Name** | **Manufactor** | **Cat.NO** | **Dilution ratio** |
| --- | --- | --- | --- |
| ACSL4 (Rabbit) | Abcam | ab155282 | 1:100 |
| GPX4(Rabbit) | Abcam | ab125066 | 1:1000 |
| SLC7A11(Rabbit) | Abcam | ab307601 | 1:1000 |
| SIRT1(Rabbit) | CST | 9475 | 1:1000 |
| AMPK(Rabbit) | Abcam | ab32047 | 1:1000 |
| p-AMPK(Rabbit) | Abcam | ab133448 | 1:1000 |
| ACC(Rabbit) | Abcam | ab45174 | 1:5000 |
| p-ACC(Rabbit) | Abcam | ab68191 | 1:5000 |
| GAPDH | Abcam | ab181602 | 1:10000 |
