# Supplementary material for: Pharmacological activation of SIRT1–AMPK by ginsenoside Rb1: a novel therapeutic strategy for pressure injury via dual suppression of ferroptosis and inflammation
Source: Front Pharmacol. 2026 Feb 17;16:1683479. doi: 10.3389/fphar.2025.1683479 (PMC12953485; doi:10.3389/fphar.2025.1683479)
Supplement: Supplementary file 4 [file Table3.docx]

**Table S3. Wound area measurements in rats from each group at different time points.**

| **Name** | **sham** | **Model** | **Rb1-L** | **Rb1-H** | **Fer-1** |
| --- | --- | --- | --- | --- | --- |
| Day 0 | 0 | 4.68±0.35 | 2.66±0.17 | 1.41±0.11 | 1.25±0.08 |
| Day 3 | 0 | 4.25±0.31 | 2.03±0.13 | 1.17±0.09 | 1.01±0.06 |
| Day 7 | 0 | 3.71±0.27 | 1.48±0.10 | 0.79±0.05 | 0.63±0.05 |
| Day 10 | 0 | 2.97±0.18 | 0.99±0.06 | 0.35±0.02 | 0.26±0.03 |
| Day 14 | 0 | 2.52±0.14 | 0.81±0.04 | 0.20±0.01 | 0.17±0.03 |

Data are presented as mean ± standard deviation (Mean ± SD).
